# Impact of COVID-19 on longitudinal ophthalmology authorship gender trends

**DOI:** 10.1007/s00417-021-05085-4

**Published:** 2021-02-03

**Authors:** Anne X. Nguyen, Xuan-Vi Trinh, Jerry Kurian, Albert Y. Wu

**Affiliations:** 1 Faculty of Medicine, McGill University, Montreal, QC Canada; 2 Department of Computer Science, McGill University, Montreal, QC Canada; 3 Department of Ophthalmology, Stanford University School of Medicine, 2370 Watson Court, Suite 200, Palo Alto, CA 94303 USA

**Keywords:** Gender, Authorship, COVID-19, Ophthalmology

## Abstract

**Background:**

The COVID-19 pandemic increased the gender gap in academic publishing. This study assesses COVID-19’s impact on ophthalmology gender authorship distribution and compares the gender authorship proportion of COVID-19 ophthalmology-related articles to previous ophthalmology articles.

**Methods:**

This cohort study includes authors listed in all publications related to ophthalmology in the COVID-19 Open Research Dataset and CDC COVID-19 research database. Articles from 65 ophthalmology journals from January to July 2020 were selected. All previous articles published in the same journals were extracted from PubMed. Gender-API determined authors’ gender.

**Results:**

Out of 119,457 COVID-19-related articles, we analyzed 528 ophthalmology-related articles written by 2518 authors. Women did not exceed 40% in any authorship positions and were most likely to be middle, first, and finally, last authors. The proportions of women in all authorship positions from the 2020 COVID-19 group (29.6% first, 31.5% middle, 22.1% last) are significantly lower compared to the predicted 2020 data points (37.4% first, 37.0% middle, 27.6% last) (*p* < .01). The gap between the proportion of female authors in COVID-19 ophthalmology research and the 2020 ophthalmology-predicted proportion (based on 2002–2019 data) is 6.1% for overall authors, 7.8% for first authors, and 5.5% for last and middle authors. The 2020 COVID-19 authorship group (1925 authors) was also compared to the 2019 group (33,049 authors) based on journal category (clinical/basic science research, general/subspecialty ophthalmology, journal impact factor).

**Conclusions:**

COVID-19 amplified the authorship gender gap in ophthalmology. When compared to previous years, there was a greater decrease in women’s than men’s academic productivity.

**Supplementary Information:**

The online version contains supplementary material available at 10.1007/s00417-021-05085-4.

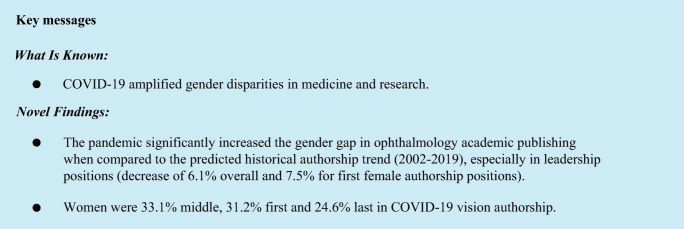

## Introduction

Clinical and basic research leadership benefit from diversity and inclusion [1, 2]. With women increasingly contributing to medical and research fields, positive trends for women in ophthalmology have been highlighted during the past decade: increasing numbers of female ophthalmology residents, higher proportions of female speakers at ophthalmology conferences, and a significant increase in women ophthalmology authors [3, 4]. Despite this progress, female underrepresentation in academic ophthalmology remains a challenge, as women’s contribution to ophthalmology authorship is well under the 50% mark. According to recent gender distribution studies in ophthalmology authorship using data from 2002 to 2018, the proportion of female authors has increased more slowly in subspecialty journals compared to general ophthalmology journals and fewer women occupy senior authorship positions [5, 6]. Female underrepresentation in academic publications, especially in senior authorship positions, is thought to lead to female faculty underrepresentation in academia [7].

The COVID-19 pandemic appears to have increased the gender gap in academic publishing [8]. A larger gender gap indicates not only a decreased diversity, but also a disproportionate decrease in female productivity. During the COVID-19 pandemic, lockdowns and social isolation measures worldwide have reshaped workplaces. Care is delivered via telemedicine when possible, researchers are forced to work from home, and children no longer attend school in person. While early studies have shown that there is a significantly higher rate of total publications in ophthalmology (likely due to decrease in clinical workload) [9], women’s productivity in medical research literature, specifically in COVID-19 studies, has been more greatly affected in comparison to men [10]. The challenges women face while working from home could explain this since household and childcare duties are largely handled by women, particularly in nations with high gender inequity [11, 12].

Gender equity improves patient care [13], innovation, and research [1]. Female doctors have been shown to engage their patients more actively in patient care compared to their male counterparts [14]. More women in healthcare allow gender-specific medical concerns to be better voiced and addressed [15]. Diversity is not only crucial in the clinical setting, but also in research teams, methods, and questions [16]. Knowing that female contributions in patient care research and innovation are beneficial, it is crucial to assess and address the increasing gender gap during the COVID-19 pandemic. To date, no study has examined the gender authorship trends in ophthalmology and vision science related to COVID-19 articles.

## Material and methods

### Data source

This observational study used publicly available source data and was ruled exempt by the Stanford University IRB/Ethics Committee (eProtocol #: 57659 - IRB 7: Registration 5136). In order to reflect work performed solely during the COVID-19 pandemic, we examined COVID-19-related ophthalmology papers, as non-COVID-19-related ophthalmology research could partially reflect work done prior to the pandemic due to delays from submission to publication [17].

**Fig. 1:**
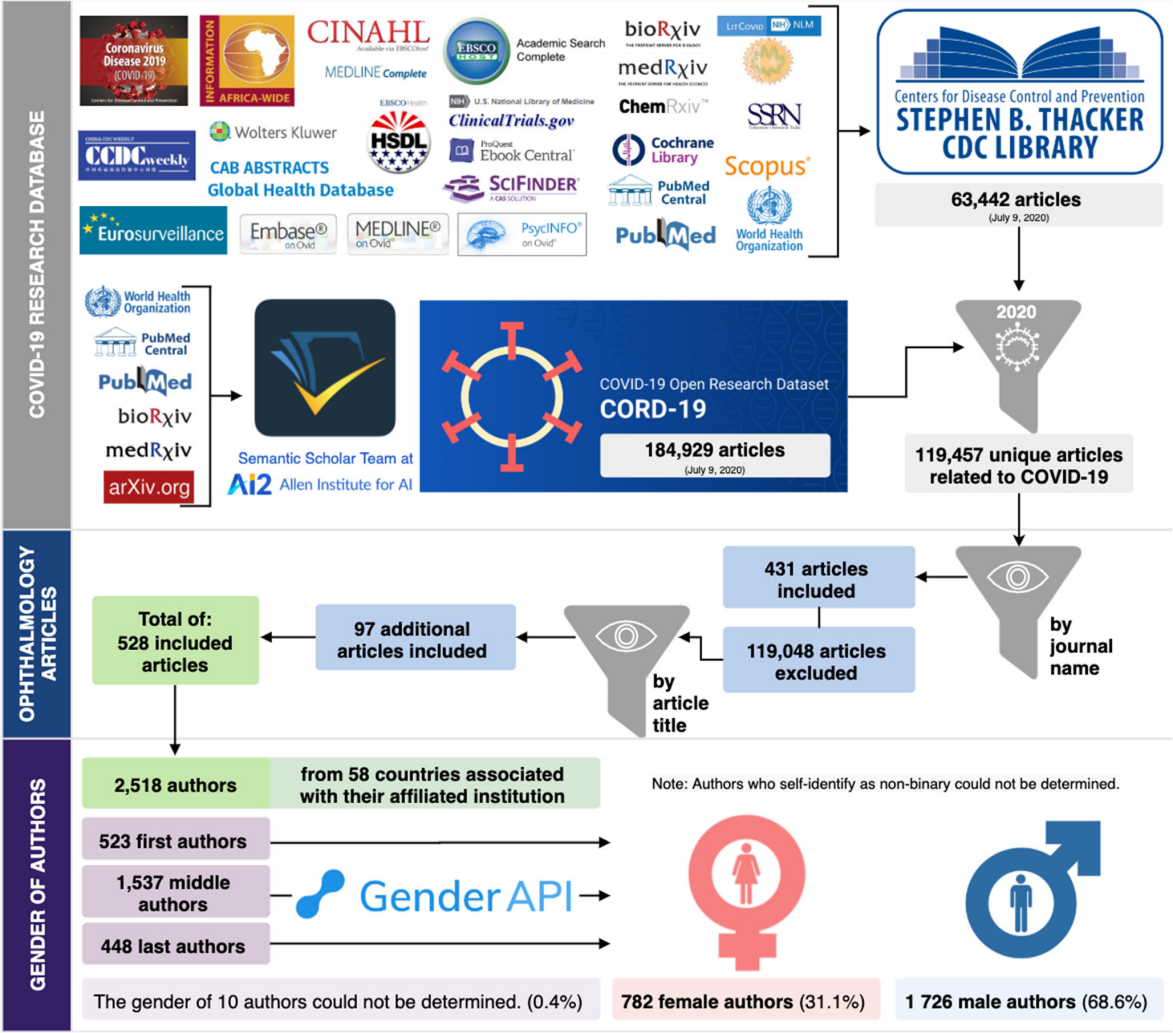
Methodology used to extract information about COVID-19 ophthalmology articles. This original figure is illustrated by author A.X.N

Data was collected from two comprehensive COVID-19 research databases (Stephen B. Thacker Centers for Disease Control and Prevention (CDC) Library and COVID-19 Open Research Dataset (CORD-19)) from January 1, 2020 to July 9, 2020. These two databases compile COVID-19-related articles from 27 major platforms, including PubMed, Scopus, and MEDLINE (Fig. 1, Supplementary Material 1). Duplication removal by title and data normalization were performed on our merged dataset.

### Article selection

To select all COVID-19 articles published in ophthalmology journals in our dataset, we used a script to extract articles from journals whose name contained regular expressions (sequence of characters defining search patterns) related to ophthalmology (e.g., opht*, ocul*, eye*, retina*) or matched the ophthalmology journals’ names listed in the 2019 Clarivate Analytics Journal Citation Reports [18]. In total, articles from 65 ophthalmology journals were included in the analyses (Supplementary Material 2).

From the remaining articles that were not published in such a journal, articles that had a title directly related to ophthalmology were included: We searched the latter for the same ophthalmology-related expressions and manually screened all article titles and abstracts. Articles that were not included mainly had titles that contained eye or vision-related idioms, such as “eye of the storm”.

Articles written by groups (e.g., La Société française d’ophthalmologie) were excluded, as author names were not displayed. Duplicate articles with titles in different languages were also removed. It should be noted that articles from all languages (including but not limited to English, French, Spanish, German, Portuguese, Chinese, and Dutch) available in the databases and that matched our filtering criteria were included in this study.

### Author information

All author information available in our COVID-19 ophthalmology article dataset were extracted with a script. Articles containing an incomplete author name (e.g., first name initial only) and/or country of affiliated institution were manually searched. Each author was assigned first (first listed author), last/senior (last listed author), and middle (other) author position. Single authors were considered first authors.

The application program interface Gender-API (https://gender-api.com/) was used to determine a person’s gender based on their first name and country of affiliated institution. For each given first name, Gender-API returns female, male, unknown (50% chance of being male or female), or undetermined (unable to identify). Specifying a person’s country improves the algorithm’s accuracy. This algorithm has been shown to be the most accurate gender assignment program (over 98% accuracy) [19, 20]. For authors with undetermined gender, we identified them by their full name and affiliated institution on professional websites (e.g., university profiles, LinkedIn, ResearchGate) and determined their gender based on their picture and descriptive paragraphs referring to them using a gender-specific pronoun (he, she, him, or her).

All authors were classified into World Bank regions (geographic location and income level) based on their affiliated institution’s location [21]. Their countries of their affiliated institutions were also associated with gender inequality index (GII) 2018 values per country based on the 2019 United Nations development program human development reports.

### Past ophthalmology authorship data

We used a Python version 3.8.6 (Python Software Foundation, Wilmington, DE, USA) script to extract author information from all past articles published in the same ophthalmology journals available on PubMed, from 1936 (earliest article’s publication year) up to December 31, 2019. Two magazines (*Optometry Times*, *Ocular Surgery News*) and four ophthalmology journals (*Chung-Hua Yen Ko Tsa Chih*, *Retina Today*, *Revista Mexicana de Oftalmología*, *Zhonghua Shiyan Yanke Zazhi*) were not available on PubMed, resulting in 58 ophthalmology journals.

To assess if COVID-19 amplified the gender gap in ophthalmology publications, we compared our 2020 COVID-19 authors’ data with the predicted 2020 ophthalmology authorship data based on the past dataset’s trend.

We also assessed COVID-19’s impact on ophthalmology publications by journal type (clinical versus basic science research, general versus subspecialty ophthalmology, 2019 impact factor versus no impact factor) by comparing our COVID-19 dataset to articles published during the same time period in 2019 (January 1, 2019 to July 9, 2019) from the past ophthalmology dataset.

### Statistical analyses

The data was analyzed with STATA/IC version 16.1 (Stata Corp, College Station, TX, USA). We calculated and compared the proportion of female authors per academic rank, geographical location (World Bank classification by region) and country income (World Bank classification by income). Fisher’s exact tests were performed to compare the proportion of female authors in COVID-related research to that of female authors in the same period from 2019 articles published in the same ophthalmology journals in the following categories: research type (clinical journals versus both clinical and basic journals), impact factor (IF) (journals with an IF versus those without), and ophthalmology type (general versus subspecialty). Linear regression was used to evaluate the trend in proportion of female authors over time. *p* values less than 0.05 were considered statistically significant.

## Results

### Overall data

After merging the two COVID-19 databases and removing duplicate articles by title, we obtained 119,457 unique articles. We extracted studies related to ophthalmology and vision science from our dataset, which resulted in a total of 528 ophthalmology-related articles (Fig. 1). These 528 articles had 2518 authors with complete first names. Gender-API determined the gender of 2485 authors, with 99% median accuracy (mean = 93.5%). We manually identified 23 authors whose gender was not returned by Gender-API. Ten authors remained unidentified, resulting in 2508 authors, which corresponded to 523 first, 1537 middle, and 448 last authors.

Out of the 408 articles published in ophthalmology journals available on PubMed, 321 (78.7%) were from clinical ophthalmology journals and 87 (21.3%) from both basic science and clinical research; 343 (84.1%) were from general ophthalmology journals and 65 (15.9%) from subspecialty journals (Supplementary Material 2).

### Overall COVID-19 ophthalmology authorship

For the 2508 authors whose gender was identified, an exact binomial test indicated that the proportion of women (31.2%) was significantly lower than the proportion of men (68.8%), *p* < .001 (Cohen’s *g* = 0.188, effect size). Women were 31.2% first, 33.1% middle, and 24.6% last authors for all COVID-19-related articles.

**Fig. 2:**
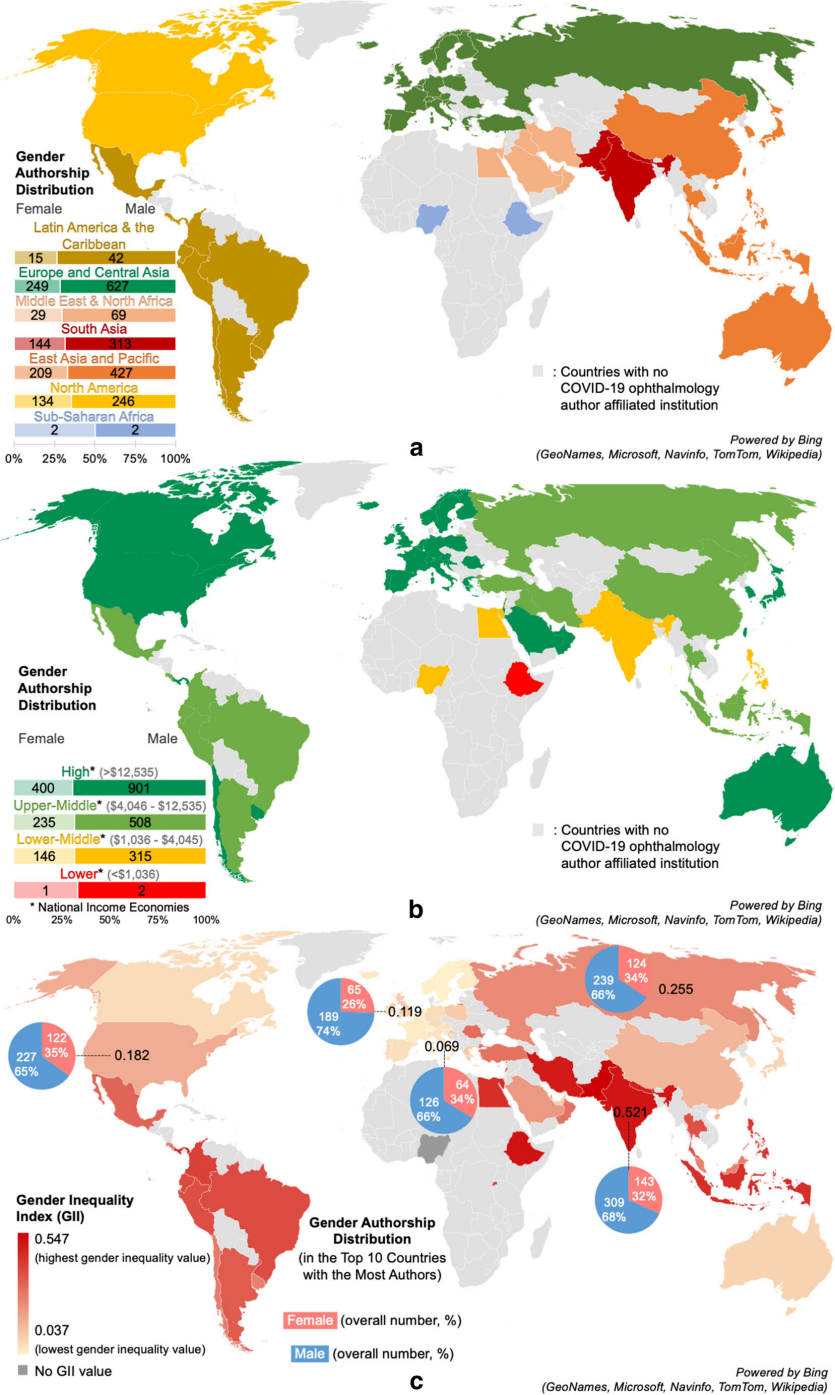
Maps representing COVID-19 ophthalmology authors by **a** World Bank classification by region, accompanied by authorship gender distribution; **b** World Bank classification by income, accompanied by authorship gender distribution*; **c** gender inequality index (GII) 2018 values from the 2019 Human Development Reports developed by the United Nations (UN) Development Program, accompanied by authorship gender distribution in the top 5 countries with the most authors from the COVID-19 ophthalmology dataset [22]. * In **b**, there are higher COVID-19 female authorship proportions in lower-income countries than high-income countries: 33.3% women from low-income countries, >31.7% women from lower-middle-income countries, >31.6% women from upper-middle-income countries, and >30.7% women from high-income countries, but these differences are not significant. This original figure is illustrated by author A.X.N

The authors are affiliated with institutions from 57 countries. The authors’ gender distributions according to their geographical regions (North America, Latin America and the Caribbean, Europe and Central Asia, East Asia and Pacific, South Asia, Middle East and North Africa, Sub-Saharan Africa) and income levels (low, lower-middle, upper-middle, high income) are shown in Fig. 2a and b.

Figure 2c shows the GII for each country associated with COVID-19 ophthalmology authors [22] and illustrates the COVID-19 ophthalmology overall authorship gender distribution in the 5 countries with the most authors (India: *N* = 452, 31.6% women; China: *N* = 363, 34.2% women; USA: *N* = 349, 35.0% women; UK: *N* = 254, 25.6% women; Italy: *N* = 200, 32.0% women).

### Longitudinal ophthalmology authorship trends

**Fig. 3:**
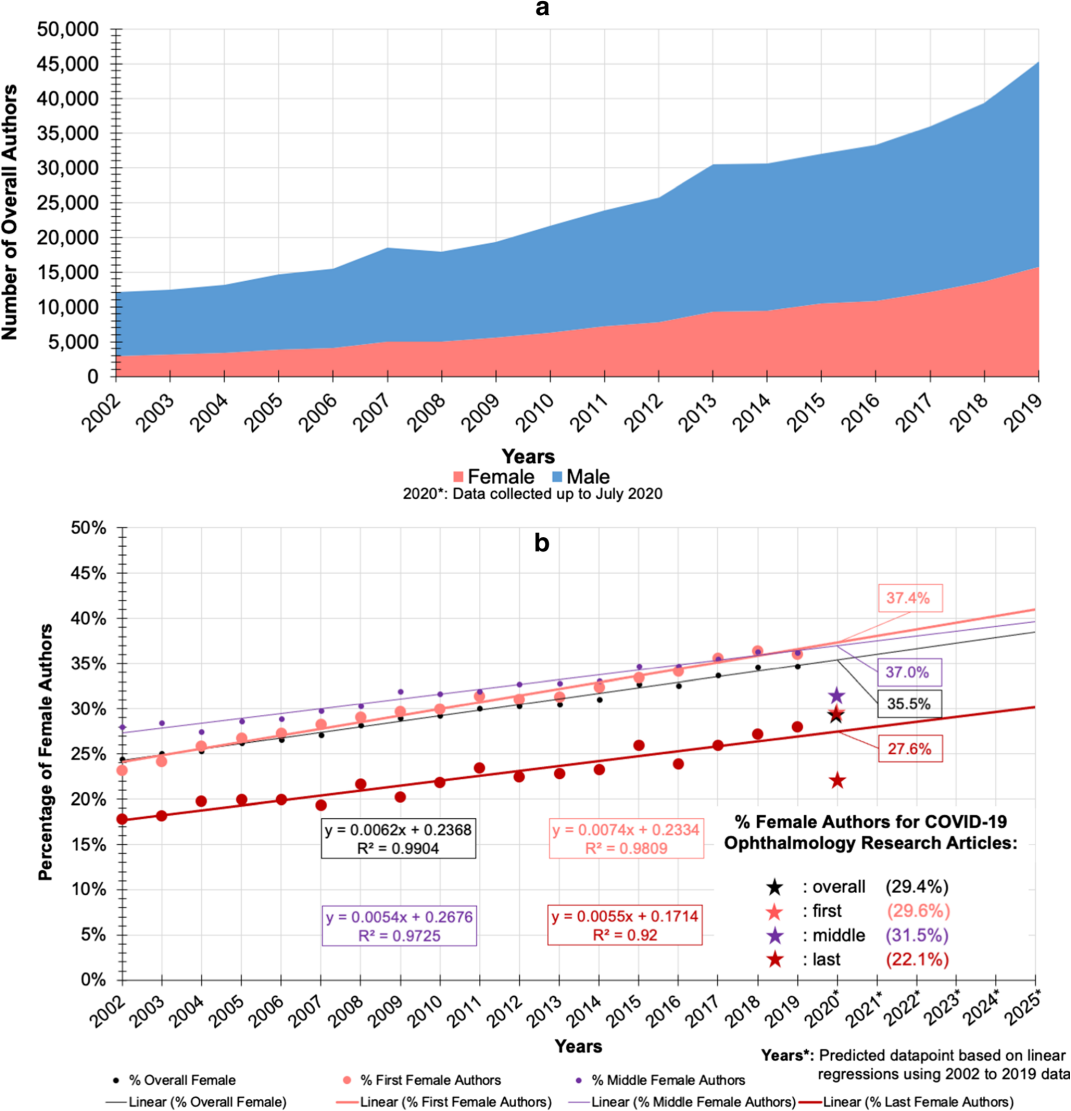
Trends in ophthalmology authorship distribution by gender from 2002 to 2019 by **a** number of overall authors and **b** percentage of female authors (overall, first, middle, last authorship positions)

A total of 444,274 authors from 1936 to 2019 were extracted. Authors from 1936 to 2001 were excluded from the linear regression calculation, as these years represented less than 1% of all authors. Authors from 2002 to 2019 were therefore used to predict gender authorship trends prior to COVID-19. Figure 3a shows the increasing author number (both male and female) over time. Figure 3a and b show an increasing number and proportion of female authors from 2002 to 2019. This tendency increases more for first female authors (*m* = 0.0074) than for all female authors (*m* = 0.0062), which also increases more than for last authors (*m* = 0.0055) and middle authors (*m* = 0.0054). When comparing the predicted 2020 proportions based on these linear regression slopes to the COVID-19 authorship data, the COVID-19 overall proportion of women was 6.1% lower than expected (29.4% instead of 35.5%). More specifically, the proportions of women in all authorship positions from the 2020 COVID-19 group (29.6% first, 31.5% middle, and 22.1% last) are significantly lower compared to the predicted 2020 data points (37.4% first, 37.0% middle, and 27.6% last) (*p* < .01) (Fig. 3b).

### Comparison to 2019 data by journal type

The 2020 COVID-19 authorship group (1925 authors, 408 articles) was compared to the 2019 group (33,049 authors, 6678 articles) based on journal category (clinical research, basic science and clinical research, general ophthalmology, subspecialty ophthalmology, and journal IF) (Table 1).

**Table 1:**
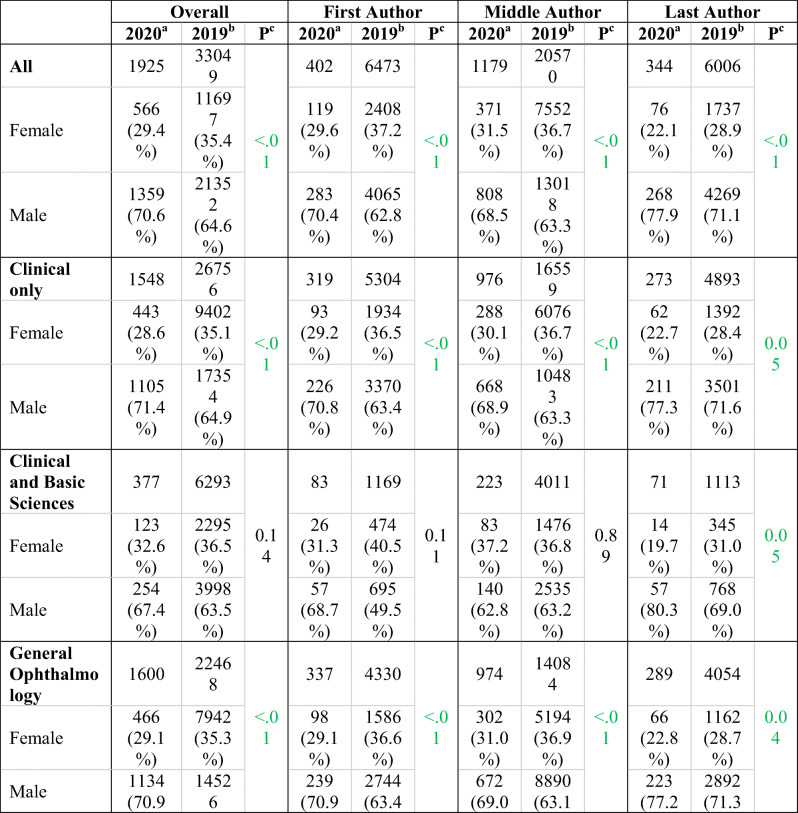

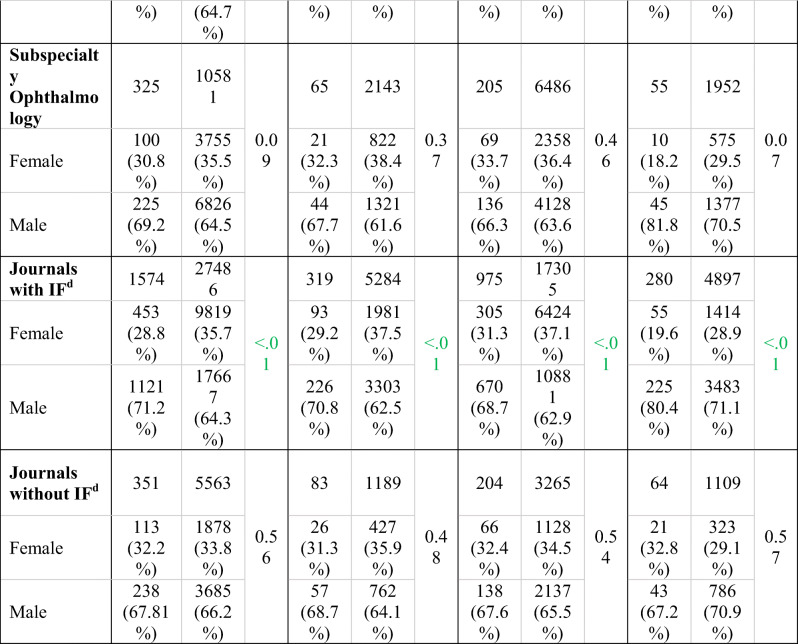
Gender distribution of articles published in ophthalmology journals found in the COVID-19 merged databases from January 1 to July 9, 2020, compared to articles published in the same journals during the same period in 2019. Gender percentages refer to overall gender, excluding unknown values. Because of unknown values, article numbers do not necessarily correspond to the sum of male and female first author

^a^COVID-19 articles in 2020

^b^Same journal articles in 2019

^c^*p* value from Fisher’s exact test; significant *p* value (*p* ≤ .05) in green

^d^Impact factor (IF) accessioned by the 2020 Clarivate Analytics Journal Citation Reports

In the 46 *clinical ophthalmology* journals, the proportion of women in the COVID-19 group (28.6%) decreased by 6.5% (*p* < .01) compared to the 2019 group (35.1%). In the 12 journals accepting both *basic science and clinical research*, the proportion of women in the COVID-19 group (32.6%) decreased by 3.9% compared to the 2019 group (36.5%), but this change was not statistically significant (*p* = 0.14).

**Fig. 4:**
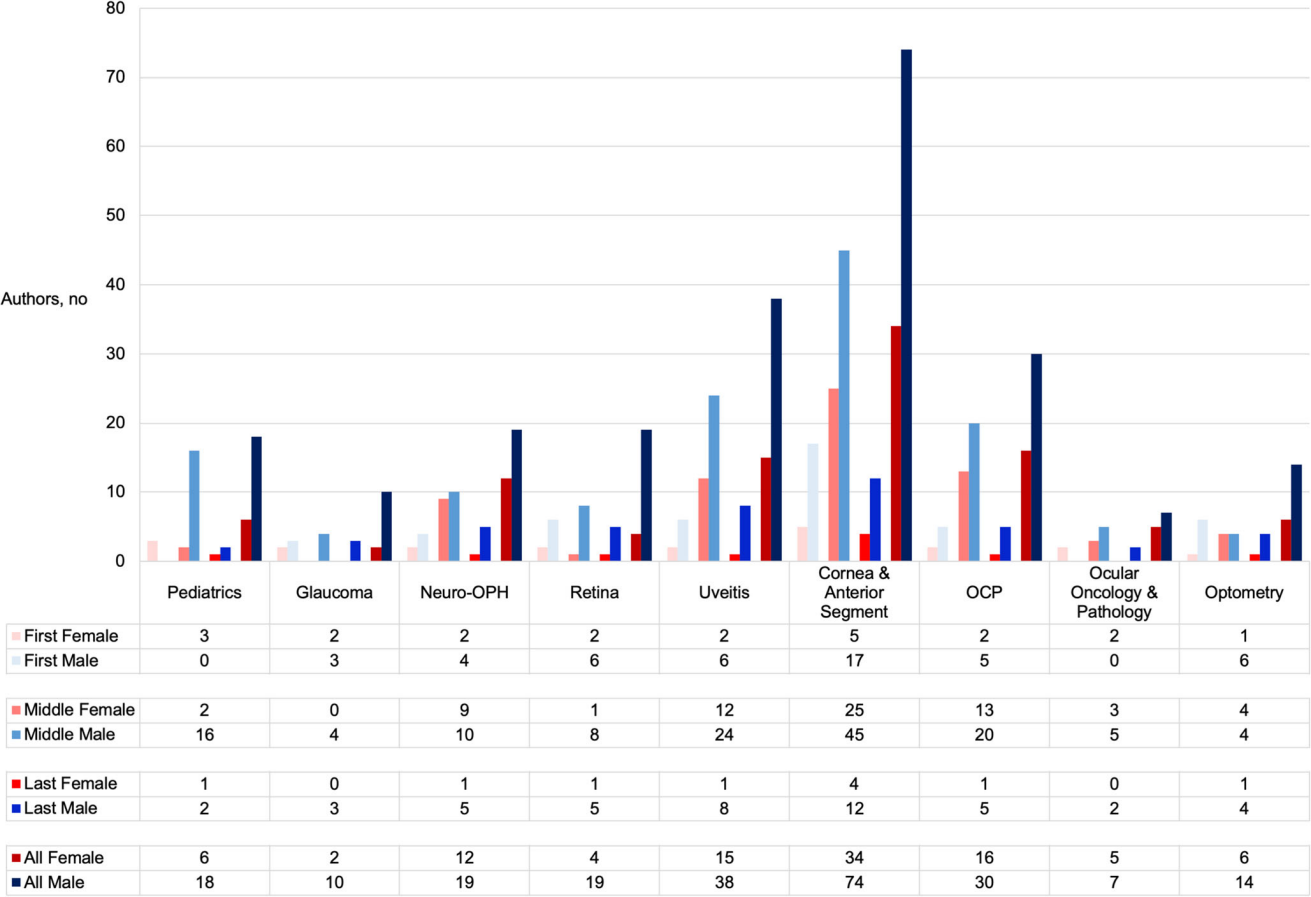
Representation of authorship gender in journals related to specific subspecialties in the COVID-19 merged databases from January 1 to July 9, 2020

In the 40 *general ophthalmology* journals, the proportion of women in the COVID-19 group (29.1%) decreased by 6.2% (*p* < .01) compared to the 2019 group (35.3%). In the 18 *subspecialty ophthalmology* journals, the proportion of women in the COVID-19 group (30.8%) decreased by 4.7% compared to the 2019 group (35.5%), but this change was not statistically significant (*p* = 0.09) (Fig. 4).

In the 34 *journals with an impact factor* (IF), the proportion of women in the COVID-19 group (28.8%) decreased by 6.9% (*p* < .01) compared to the 2019 group (35.7%). In the 25 *journals without an IF*, the proportion of women in the COVID-19 group (32.2%) decreased by 1.6% compared to the 2019 group (33.8%), but this change was not statistically significant (*p* = 0.06).

## Discussion

### Fewer women overall and in first, middle, and last authorship positions in COVID-19 ophthalmology research publications

In this study, women did not exceed the 40% mark in any authorship position and category. Overall women represented 33.1% middle, 31.2% first, and 24.6% last authorship positions. Women were most likely to be middle, followed by first and then last author in all categories, including clinical, basic and clinical science, general ophthalmology, and subspecialty journals. Conventionally, the first listed author is considered the lead investigator, the last author is the senior (or corresponding) author, and the remaining authors are middle authors. First and last authors are considered leadership positions [23]. This study shows that women occupy fewer research leadership positions in COVID-19-related ophthalmology studies than their male colleagues.

These results are in line with early findings in other medical fields, notably public health, internal medicine, and radiology [8, 24]. The pandemic has brought challenges to all researchers, due to ongoing social isolation measures leading to predominant work from home. With limited access to childcare, early and mid-career women are particularly affected by COVID-19 [25]. Late-career women are also affected, as their age group may predispose them to retire early due to circumstances surrounding the pandemic [26]. Studies examining the gender gap in academia have proposed multiple theories: the historical workplace marginalization of women, the lack of female research leadership opportunities, and unblinded peer review bias [27]. While our observational results cannot conclusively correlate COVID-19 challenges with a decrease in female authorship, they suggest that women are unequally burdened by societal changes occurring during the pandemic.

### COVID-19 ophthalmology authorship compared to previous ophthalmology authorship

COVID-19 has increased the authorship gender gap in ophthalmology. The gap between the proportion of female authors in COVID-19 ophthalmology research and the predicted 2020 proportion of female authors based on the trend of previous years (2002–2019) is 6.1% for overall authors, 7.8% for first authors, and 5.5% for last and middle authors (Fig. 3b).

A gap in first authorship positions is found when comparing COVID-19 ophthalmology authorship to the 2019 comparator group in clinical journals (significant difference of 7.3%), clinical and basic science journals (9.2% difference), general ophthalmology journals (significant difference of 7.5%), and subspecialty journals (6.1% difference). The differences are also high for senior authorship position in clinical journals (significant difference of 5.7%), clinical and basic science journals (significant difference of 11.3%), general ophthalmology journals (significant difference of 5.9%), and subspecialty journals (11.3% difference). The gap is therefore larger for women occupying leadership (first and last) positions during COVID-19.

### Limitations

The main study limitation was that the authors did not self-identify their gender, which would have confirmed gender assignments and allowed for a non-binary gender spectrum. Indeed, we had to use an imperfect gender predictive algorithm, albeit correct 98% of the time (Gender-API’s estimated accuracy). Furthermore, while we acknowledge that gender exists on a spectrum and is socially produced, we were constrained by Gender-API’s binary gender output.

### Possible solutions to address female authorship underrepresentation

Implicit and unconscious biases could be responsible for this 2020 gender gap. These biases can start to be overcome through adequate management training, mentorship, and sponsorship, which are strategies to promote women’s place in academia [28].

On a larger scale, these biases can be reinforced by national, regional, local, and institutional policies. Female underrepresentation seems to occur to a greater degree in countries with high gender inequality indices: India with a high GII value (0.501) has 31.6% women authors, while the USA with a lower GII value (0.182) has 35.0% women authors (Fig. 2c). When excluding geographical regions with a small number of authors (e.g., Sub-Saharan Africa with 4 authors), a similar trend can be noted: North America (lower GII, 35.3% women) has a higher proportion of female authors than South Asia (high GII, 31.5% women) and Latin American and the Caribbean (high GII, 26.3% women) (Fig. 2a).

COVID-19 may exacerbate these pre-existing challenges for women. Reliable access to childcare has been listed as a major source of anxiety for healthcare professionals, especially with school closures and increased work hours [29]. During the pandemic’s first peak of cases, countries like the UK (GII = 0.119) and Canada (GII = 0.083) provided temporary emergency childcare to essential workers. These social policies supporting workers, in addition to long-term job security and workplace re-entry support plans, can be particularly beneficial to early and mid-career women [30].

Public policies that help bridge the gender gap in research involve ensuring equal pay, granting basic legal rights and reforms (e.g., right to education, freedom of choice, countering practices leading to sex imbalance at birth), and efficiently implementing and encouraging couples to take advantage of shared parental leaves [31]. Housework and childcare more often are the female partner’s responsibility (when there is one), and a shift toward balanced sharing of these duties would allow for greater female academic productivity and career advancement [32].

Publication and submission processes should be examined and potentially reshaped [33]. While double masking may reduce reviewer bias, a recent study demonstrates that the double-blind review process (where authors nor reviewers did not know each other’s identity) did not increase the incidence of female authorship [34]. An alternative solution could be to disclose and monitor author and reviewer genders by editorial teams, which would actively encourage gender-diverse teams [10]. A more aggressive stance could be the use of quotas, which have been proven to be an effective solution to diminish demographic gaps in politics and economics [35]. Quotas could lead to an increase in female leadership positions, such as more female academics and more women in the senior authorship position [36]. The submission, review, and publication process has been shown to have bias and has only slowly evolved over the past 50 years. Further efforts are needed to ensure that this process is fair, based on academic merit, and gender-blind [37].

## Conclusion

Despite an increase in female academic representation in ophthalmology in the past decades, our study shows that COVID-19 has reversed this progression: Women’s contribution to COVID-19 ophthalmology scholarship during the pandemic was significantly lower than expected, especially in leadership positions. To help guide institutional policies toward workplace equity, future studies should robustly identify and monitor the pandemic’s impact on targeted age, ethnic/racial and non-binary gender groups [24]. While the pandemic has reshaped workspaces and increased challenges in authorship for women, there are many options to try to bridge the gender gap by first highlighting these findings and then implementing solutions to address systemic disadvantages women now face.

## Supplementary information

ESM 1 (DOCX 37 kb)

ESM 2 (DOCX 33 kb)
