# Supplementary material for: Impact of COVID-19 on longitudinal ophthalmology authorship gender trends
Source: Graefes Arch Clin Exp Ophthalmol. 2021 Feb 3;259(3):733–44. doi: 10.1007/s00417-021-05085-4 (PMC7857347; doi:10.1007/s00417-021-05085-4)
Supplement: Supplementary file 1 — (DOCX 37 kb) [file 417_2021_5085_MOESM1_ESM.docx]

**SUPPLEMENTARY MATERIAL 1**

**Database (Figure 1)**

All articles in the daily updated COVID-19 research databases compiled by the Stephen B. Thacker Centers for Disease Control and Prevention (CDC) Library and COVID-19 Open Research Dataset (CORD-19) were examined in this study.^1, 2^ The Stephen B. Thacker CDC Library systematically searches open access databases, including ClinicalTrials, PubMed Central, PubMed Medline, LitCovid (NLM), CDC Novel Coronavirus page, EuroSurveillance, China CDC MMWR, Homeland Security Digital Library, bioRxiv, medRxiv, chemRxiv, SSRN, and the World Health Organisation (WHO) databases (Virtual Health Library, WHO Novel Coronavirus Page), as well as databases requiring authentication, such as Ovid databases (Medline, Embase, CAB Abstracts, Global Health, PsychInfo), the Cochrane Library, Scopus, EBSCO databases (Academic Search Complete, Africa Wide Information, CINAHL), ProQuest Central (ProQuest), and SciFinder (CAS). After extracting relevant COVID-19 research articles, the Stephen B. Thacker CDC Library deduplicates them using EndNote. CORD-19 is a larger open dataset that collects and deduplicates papers and preprints from multiple sources, such as PubMed Central, PubMed, the World Health Organization’s COVID-19 Database, bioRxiv, medRxiv, and arXiv, through Semantic Scholar. Both datasets were merged in order to generate the most comprehensive COVID-19 research database. Data filtering, following the CDC’s detailed search strategy for the systematic literature review on COVID-19, was performed on this combined dataset to remove articles examining coronaviruses other than COVID-19.^2^ After normalizing the dataset (lowercasing, punctuation removal, and trailing spaces removal), additional filtering was performed to remove duplicates and retain articles with the most complete author information.^3^ An algorithm based on the Levenshtein distance was used to calculate the percentage of similarity between titles. Titles that were 90% or more similar to another title were removed. The threshold was chosen to account for discrepancies in character encoding. Article deduplication was not based on digital object identifiers (DOIs, unique and standardized article identifiers) because the same preprints versions have slightly different DOIs.

References

1. Lu Wang L, Lo K, Chandrasekhar Y, Reas R, Yang J, Eide D *et al.* CORD-19: The Covid-19 Open Research Dataset. *ArXiv* 2020.

2. COVID-19 Research Articles Downloadable Database. *Centers for Disease Control and Prevention*. Available at: <https://www.cdc.gov/library/researchguides/2019novelcoronavirus/researcharticles.html>. Accessed July 9, 2020.

3. Yujian L, Bo L. A normalized Levenshtein distance metric. *IEEE Trans Pattern Anal Mach Intell* 2007; **29**(6)**:** 1091-1095.
