# Supplementary material for: Impact of COVID-19 on longitudinal ophthalmology authorship gender trends
Source: Graefes Arch Clin Exp Ophthalmol. 2021 Feb 3;259(3):733–44. doi: 10.1007/s00417-021-05085-4 (PMC7857347; doi:10.1007/s00417-021-05085-4)
Supplement: Supplementary file 2 — (DOCX 33 kb) [file 417_2021_5085_MOESM2_ESM.docx]

**SUPPLEMENTARY MATERIAL 2.**

**Supplementary Table 1.** Ophthalmology Journals in the COVID-19 merged research database (CDC COVID-19 database and CORD-19)

| **Journal Name** | **Impact Factor^19^** | **Ophthalmology Type** | | **Research Type** | | **Articles, no. (%)** | |  |
| --- | --- | --- | --- | --- | --- | --- | --- | --- |
| Acta Ophthalmologica | 3.362 | general | | clinical | | 15 (2.8%) | |  |
| American Journal of Ophthalmology Case Reports | None | general | | clinical | | 2 (0.4%) | |  |
| American Journal of Ophthalmology | 4.013 | general | | clinical | | 5 (1.0%) | |  |
| Archivos de la Sociedad Espanola de Oftalmologia | None | general | | clinical | | 15 (2.8%) | |  |
| Arquivos brasileiros de oftalmologia | 0.617 | general | | clinical | | 2 (0.4%) | |  |
| Asia Pac J Ophthalmol (Phila) | None | general | | clinical | | 3 (0.6%) | |  |
| BMJ Open Ophthalmol | None | general | | basic science and clinical | | 2 (0.4%) | |  |
| British Journal of Ophthalmology | 3.611 | general | | clinical | | 8 (1.5%) | |  |
| British Journal of Visual Impairment | None | general | | clinical | | 1 (0.2%) | |  |
| Canadian Journal of Ophthalmology | 1.369 | general | | clinical | | 2 (0.4%) | |  |
| Chung-Hua Yen Ko Tsa Chih [Chinese Journal of Ophthalmology]* | None | general | | clinical | | 2 (0.4%) | |  |
| Clinical & experimental optometry | 1.918 | optometry | | basic science and clinical | | 3 (0.6%) | |  |
| Community Eye Health | None | general | | clinical | | 1 (0.2%) | |  |
| Contact Lens & Anterior Eye | 2.578 | cornea and anterior segment | | basic science and clinical | | 11 (2.1%) | |  |
| Cornea | 2.215 | cornea and anterior segment | | clinical | | 3 (0.6%) | |  |
| Current Opinion in Ophthalmology | 2.983 | general | | clinical | | 1 (0.2%) | |  |
| Current eye research | 1.754 | general | | basic science and clinical | | 1 (0.2%) | |  |
| Current Ophthalmology Reports | None | general | | clinical | | 2 (0.4%) | |  |
| Cutaneous and ocular toxicology | 1.385 | general | | basic science and clinical | | 1 (0.2%) | |  |
| Der Ophthalmologe | 0.698 | general | | clinical | | 15 (2.8%) | |  |
| European Journal of ophthalmology | 1.642 | general | | clinical | | 3 (0.6%) | |  |
| Eye | 2.455 | general | | clinical | | 52 (9.9%) | |  |
| Eye Reports | None | general | | clinical | | 3 (0.6%) | |  |
| Eye and Vision | 2.241 | general | | clinical | | 2 (0.4%) | |  |
| Graefes Archive for Clinical & Experimental Ophthalmology | 2.396 | general | | basic science and clinical | | 32 (6.1%) | |  |
| Indian Journal of Ophthalmology | 1.250 | general | | clinical | | 81 (15.3%) | |  |
| International Journal of Retina and Vitreous | None | retina | | clinical | | 1 (0.2%) | |  |
| International Journal of Ophthalmology | 1.330 | general | | clinical | | 1 (0.2%) | |  |
| International ophthalmology | 1.314 | general | | clinical | | 2 (0.4%) | |  |
| International Eye Science | None | general | | basic science and clinical | | 5 (1.0%) | |  |
| International ophthalmology clinics | None | general | | clinical | | 1 (0.2%) | |  |
| Journal of AAPOS | 1.339 | pediatrics | | clinical | | 3 (0.6%) | |  |
| Journal of Cataract and Refractive Surgery | 2.689 | cornea and anterior segment | | clinical | | 3 (0.6%) | |  |
| Journal of Current Glaucoma Practice | None | glaucoma | | clinical | | 1 (0.2%) | |  |
| Journal of Optometry | None | optometry | | clinical | | 2 (0.4%) | |  |
| JAMA Ophthalmology | 6.198 | general | | clinical | | 4 (0.8%) | |  |
| Journal francais d'ophtalmologie | 0.636 | general | | clinical | | 21 (4.0%) | |  |
| Journal of glaucoma | 1.992 | glaucoma | | clinical | | 4 (0.8%) | |  |
| Journal of Neuro-Ophthalmology | 2.513 | neuro-ophthalmology | | clinical | | 6 (1.1%) | |  |
| Journal of Ocular Pharmacology and Therapeutics | 1.925 | general | | basic science and clinical | | 1 (0.2%) | |  |
| Journal of Visual Impairment & Blindness | None | general | | clinical | | 2 (0.4%) | |  |
| Klinische Monatsblatter fur Augenheilkunde | 0.605 | general | | clinical | | 1 (0.2%) | |  |
| Medical hypothesis, discovery & innovation ophthalmology journal | None | general | | clinical | | 2 (0.4%) | |  |
| Middle East Afr J Ophthalmol | None | general | | clinical | | 1 (0.2%) | |  |
| Ocular Oncology and Pathology | None | ocular oncology and ocular pathology | | basic science and clinical | | 2 (0.4%) | |  |
| Ocular immunology and inflammation | 2.112 | uveitis | | basic science and clinical | | 9 (1.7%) | |  |
| Ocular Surface | 12.336 | cornea and anterior segment | | basic science and clinical | | 5 (1.0%) | |  |
| Ocular Surgery News** | None | general | | clinical | | 3 (0.6%) | |  |
| Oman Journal of Ophthalmology | None | general | | clinical | | 2 (0.4%) | |  |
| Ophthalmic plastic and reconstructive surgery | 1.331 | oculoplastics | | clinical | | 3 (0.6%) | |  |
| Ophthalmology | 8.470 | general | | clinical | | 22 (4.2%) | |  |
| Ophthalmology and therapy | None | general | | clinical | | 11 (2.1%) | |  |
| Ophthalmology. Retina | None | retina | | clinical | | 3 (0.6%) | |  |
| Optometry and vision science | 1.458 | optometry | | clinical | | 1 (0.2%) | |  |
| Optometry Times** | None | optometry | | clinical | | 1 (0.2%) | |  |
| Orbit | None | oculoplastics | | clinical | | 4 (0.8%) | |  |
| RETINA-The Journal of Retinal and Vitreous Diseases | 3.649 | retina | | clinical | | 1 (0.2%) | |  |
| Retina Today* | None | retina | | clinical | | 2 (0.4%) | |  |
| Retina-Vitreus/Journal of Retina-Vitreous | none | retina | | basic science and clinical | | 1 (0.2%) | |  |
| Revista Mexicana de Oftalmología* | None | general | | clinical | | 1 (0.2%) | |  |
| Spektrum Augenheilkd | None | general | | clinical | | 1 (0.2%) | |  |
| Survey of Ophthalmology | 4.195 | general | | clinical | | 1 (0.2%) | |  |
| Turkish Journal of ophthalmology | None | general | | clinical | | 1 (0.2%) | |  |
| [Zhonghua yan ke za zhi] Chinese journal of ophthalmology | None | general | | basic science and clinical | | 15 (2.8%) | |  |
| Zhonghua Shiyan Yanke Zazhi/Chinese Journal of Experimental Ophthalmology* | None | general | | basic science and clinical | | 12 (2.2%) | |  |
| Non-ophthalmology journal* | NA | | NA | | NA | | 98 (18.6%) | |
| TOTAL | NA | | NA | | NA | | 528 (100.0%) | |

* Journals that were not included in the 2019-2020 comparison

** Magazines that were not included in the 2019-2020 comparison
